# Supplementary material for: Ingroup favoritism and outgroup derogation in intergenerational cooperation
Source: Commun Psychol. 2025 Jun 6;3:89. doi: 10.1038/s44271-025-00272-z (PMC12144128; doi:10.1038/s44271-025-00272-z)
Supplement: Supplementary file 2 — Supplemental Information [file 44271_2025_272_MOESM2_ESM.docx]

**Supplemental Material for**

**Intergenerational Intergroup Cooperation: Future ingroup favoritism and outgroup derogation in the minimal and natural group contexts**

Hirotaka Imada^1,2^, Yukako Inoue^3^, Alice Yamamoto-Wilson^4^,

Tatsuyoshi Saijo^5,6^, Nobuhiro Mifune^2,7^

^1^Department of Psychology, Royal Holloway, University of London, Egham, the United Kingdom.

^2^Research Institute for Future Design, Kochi University of Technology, Kochi, Japan.

^3^Department of Social Psychology, Yasuda Women’s University, Hiroshima, Japan.

^4^Independent Researcher, Tokyo, Japan

^5^Kyoto University of Advanced Science, Kyoto, Japan.

^6^Research Institute for Humanity and Nature, Kyoto, Japan.

^7^School of Economics & Management, Kochi University of Technology, Kochi, Japan.

Corresponding author: Hirotaka Imada, Department of Psychology, Royal Holloway, University of London, Egham, Surrey, TW20 0EX, UK (email: [Hirotaka.Imada@rhul.ac.uk](mailto:Hirotaka.Imada@rhul.ac.uk)).

**S1: Robust regressions (Study 1)**

We conducted Leven’s test for homogeneity of variance on reputational concern (*F*(2, 1390) = 2.19, *p* = .11), legacy motivation (*F*(2, 1390) = 0.92, *p* = .40), affinity (*F*(2, 1390) = 0.85, *p* = .43), and responsibility (*F*(2, 1390) = 0.74, *p* = .48). Thus, the assumption of homoscedasticity is met for the four continuous dependent variables. We also conducted Shapiro-Wilk normality test on reputational concern (*W* = 0.97, *p* < .001), legacy motivation (*W* = 0.98, *p* < .001), affinity (*W* = 0.97, *p* < .001), and responsibility (*W* = 0.98, *p* < .001). Thus, the assumption of normality was violated. We therefore conducted robust regression analyses using rlm() from {MASS} package. Consistemtly with the results from the normal regression analyses, Contrast 1 (ingroup vs. control) had a significant effect on legacy motivation (*b* = 0.24, *p* = .01), affinity (*b* = 0.26, *p* = .006), and responsibility (*b* = 0.44, *p* < .001), but not on reputational concern (*b* = 0.05, *p* = .67). Contrast 2 (outgroup vs. control) had a significant effect on reputational concern (*b* = -0.26, *p* = .01), but not on legacy motivation (*b* = -0.03, *p* = .71), affinity (*b* = 0.002, *p* = .98), and responsibility (*b* = -0.02, *p* = .87).

**S2: Robust regressions (Study 2)**

We conducted Leven’s test for homogeneity of variance on reputational concern (*F*(2, 1802) = 5.58, *p* = .004), legacy motivation (*F*(2, 1802) = 0.91, *p* = .40), affinity (*F*(2, 1802) = 12.11, *p* < .001), and responsibility (*F*(2, 1802) = 6.47, *p* = .002). Thus, the assumption was violated except for legacy motivation. We also conducted Shapiro-Wilk normality test on reputational concern (*W* = 0.99, *p* < .001), legacy motivation (*W* = 0.99, *p* < .001), affinity (*W* = 1.00, *p* < .001), and responsibility (*W* = 0.99, *p* < .001). Thus, the assumption of normality was violated. Where both assumptions are violated, we conducted robust regression analyses with robust standard errors using {sandwich} and {lmtest} packages. For legacy motivation, since the homoscedasticity was not violated, we used the same approach as in S1. For legacy motivation, consistently with the results from the normal regression analysis, Contrast 1 was not significant (*b* = 0.16, *p* = .08), but Contrast 2 was (*b* = -0.25, *p*  = .006). For reputational concern, Contrast 1 was not significant (*b* = 0.14, *p* = .08), but Contrast 2 was (*b* = -0.23, *p* = .002). For affinity, Contrast 1 was not significant (*b* = 0.29, *p* < .001), but Contrast 2 was (*b* = -0.30, *p* < .001). For responsibility, both Contrast 1 (*b* = 0.10, *p* = .14) and Contrast 2 (*b* = -0.17, *p* = .01) were significant. Overall, the robust regression analyses offered consistent results with the preregistered regression analyses.
